# Failure Mechanisms and Reinforcing Modes of Ply Splice Fiber-Reinforced Composite Laminates under Tensile Load

**DOI:** 10.3390/ma12182912

**Published:** 2019-09-09

**Authors:** Meng Zhu, Dingding Chen, Qigao Hu

**Affiliations:** 1 College of Aerospace Science and Engineering, National University of Defense Technology, Changsha 410000, China; 2 College of Military Basic Education, National University of Defense Technology, Changsha 410000, China

**Keywords:** carbon fiber-reinforced plastic, ply splice, failure mechanism, finite element model, cohesive zone model, reinforcement

## Abstract

To fabricate large-scale or unusually shaped composite structures, pieces of fabric plies can be spliced to match size and shape requirements, forming ply splice structures. The junction of different plies can be considered as a defect in the resulting composite material, affecting the overall mechanical properties. In this paper, unidirectional carbon fiber-reinforced plastic (CFRP) with ply splices was used as a research object to study these potential material defects. The effects of ply splices at different positions on the tensile properties of CFRP and the coupling between position of ply splicing were analyzed. Simultaneously, a finite element model was established to analyze the damage evolution, in which a continuous damage model and a cohesive zone model were used to describe the damage of the composite and interface layers, respectively. The model results were in good agreement with observed experimental results. Our results showed that there were three main factors for this failure mechanism: boundary effects, whether the ply splices were independent, or whether they were close to each other. In short, when two ply splices were located at the edge or independent of each other, the failure mode was first delamination and then fiber fracture, and the tensile strength was high. However, when the two ply splices were close to the edge or close to each other, the failure mode was first local fiber fracture and then delamination damage, and the resulting tensile strength was low. Finally, different reinforcement methods to improve the tensile properties of composites were adopted for the splicing layers at different positions through the analysis via model simulation. The two-side patch repair method was used to reinforce the ply splices on or near the edge. Additionally, increasing the toughness of the adhesive layer was used to reinforce the ply splices that were inside the material. These results showed that the tensile strength was enhanced by these two methods of reinforcement, and the initial damage load was especially increased.

## 1. Introduction

As a typical representative of advanced reinforced polymers, carbon fiber-reinforced polymers (CFRP) have been widely used in aerospace and other fields [1,2]. However, as the size of reinforced polymer structures increases, new problems such as inadequate length or width of a reinforced polymer arise. To address these problems, shearing and splicing have been utilized for engineering complicated shapes to avoid wrinkles during the plying process or to meet specific dimensional requirements. When a ply splice occurs, it inevitably introduces defects at the splicing positions, which may pose serious safety hazards when bearing force during use.

At present, there are relatively few studies directly related to spliced structures. Jia et al. [3,4,5,6,7,8,9,10,11] conducted a series of tests on the properties of the spliced structure of carbon fiber-reinforced resin-based polymer CFRP laminates. They tested the tensile strength, the overall and local stiffness, and the in-plane shear strength of the spliced layup materials, carried out a numerical simulation of the material, and obtained results indicating that the splicing stress concentration was high and that the failure mode of the ply splice laminates was due to interlaminar shear failure and fiber breakage. In the above study, the macroscopic mechanical properties of a laminate with ply splicing were carefully studied. However, as a defect, there is still a lack of research, from the view of micro-mechanics, on how junctions affect the mechanical properties of a ply spliced laminate composite. Chen et al. [12] tested the tensile properties of three unidirectional carbon fiber-reinforced laminate polymers (CFRP) with asymmetric splicing structures and analyzed the failure characteristics of these structures during the tensile process. They concluded that the shear stress and lateral tensile stress on the spliced structure during the tensile process will lead to an initial failure and that the splicing of the layered structure improved the overall strength. Huang et al. [13] tested the dislocation of ply splice laminates, measuring the tensile strength and stiffness, observed the failure characteristics of the resulting fractures and the stress distribution of the surface layer, and studied the load transfer and failure mechanism of such material. Their results led to the conclusion that the material was transferred by the matrix in shear mode and the small range of strength and stiffness increased with increasing splicing distance. Based on these results, it can be inferred that the effect on the mechanical properties of coupling between nodes of a splicing structure should also be paid attention to during a mechanical analysis of a spliced structure.

Finite element analysis is an effective method for mechanical analysis. In the fracture process of ply splice structures, the in-layer and interlaminar fracture should be carefully simulated. For in-layer failure criteria, many scholars have studied the initiation and extension of damage of composite structures with different finite element models [14,15,16,17], in which the 3D-Hashin criterion is one of the most widely used models. In terms of tensile test, Chen et al. [18] conducted tensile test on carbon fiber composite laminates without and with cracks (fracture cracks with different lengths), and employed 3D-Hashin criterion to simulate the fracture cracks of composite laminates and predict the failure load of laminates containing damage. By comparing the simulated value with the experimental ones, the two are almost identical, which verifies the reliability of the model. For the simulation of interlaminar fracture, the cohesive zone model (CZM) is normal method. CZM uses a bilinear constitutive equation to describe the whole process from initiation to extension, which is very suitable to simulate the interlayer failure of composite materials. Yang and Cox [19] successfully simulated the layering of porous composite laminates by CZM model. Camanho et al. [20,21,22] simulated delamination damage of the double contilever beam (DCB) test, the end notched flexure (ENF) test and the mixed mode bending (MMB) test specimens by CZM model, reinforced rib structure and thick composite pieces, and the results were verified by experiments. Cristobal [23,24] et al. developed a finite element model to simulate the impact behaviors of composite laminates with cohesive models. 

As mentioned above, the ply splicing induces stress concentrations, making a structure weaker. However, when a ply splice is close to another one or near the surface of the material, the coupling effects will further decrease the strength. In practice, for a large scale and complex shape composite structure, almost all the layers need ply splices. Consequently, a deep understanding of fracture mechanism of ply splice structure, including the coupling effects, is helpful to design a high strength structure. The tensile properties of these CFRPs were tested through 0° tensile experiment, and the effect of splicing positions on the mechanical properties of CFRP materials and the coupling between splicing points were analyzed. These data provided a basis for subsequent study of the influence of multiple splicing points on the structural strength at different locations. Simultaneously, the structure was simulated using the finite element method with 3D-Hashin failure criterion, and a progressive damage model and cohesive zone model were established to describe the damage evolution and adhesive failure of the structure. In this paper, the failure mechanism, especially the coupling effects, were studied through experiments and simulation works. Based on the fracture mechanism, some reinforcing methods were proposed and proved effective.

## 2. Experimental

### 2.1. Materials and Specimens

**Figure 1:**
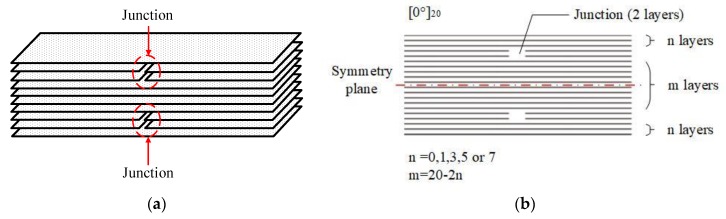
Research object. (**a**) sketch of the studied ply splice structure; (**b**) the case of [0°]_20_.

To clarify the effects of ply splicing on tensile properties, including coupling effects between splicing points and boundary effects, two junctions were designed in different locations of a laminate, as shown in Figure 1a. In order to ensure the symmetry of the structure, the samples were designed with 20 layers, which included two ply splices inside with two layers and an interlayer with different layers along the direction of thickness. When the junction is close to the surface, or even on the surface, the boundary effects will be serious; making the junctions close to the middle surface can highlight the coupling effects. Consequently, five kinds of samples with different vertical distances between the two-ply splices were designed. The numbers of lateral continuous fiber layers between the two-ply splices were 16, 14, 10, 6, and 2, as shown in Figure 1b. The thickness of the finished laminate was about 2.5 mm. The form of the ply splice is shown in Figure 1. The above-mentioned five unidirectional carbon fiber-laminated plates were marked as S0/m, S0/16, S0/14, S0/10, S0/6, and S0/2, where 0 represents the laminate direction, and m represents the number of continuous layers between the two ply splices. In order to conduct comparative experiments, a group of CFRP samples without splicing structures and with 20 layers were added for comparative analysis, and these were marked as D0.

### 2.2. Tensile Tests

**Figure 2:**
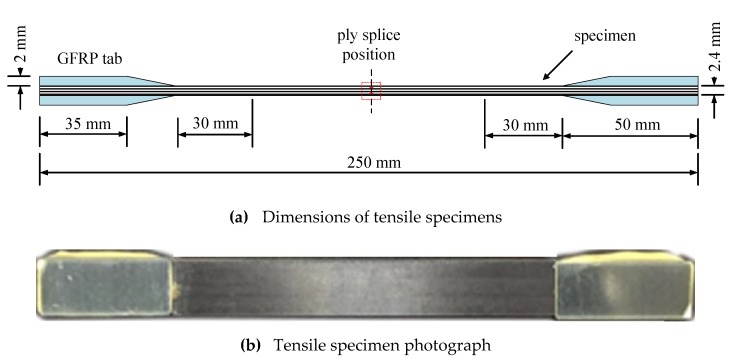
Tensile specimen. (**a**) Dimensions of tensile specimens; (**b**) Tensile specimen photograph.

With reference to ASTM D3039 [25], tensile properties were studied using static tensile tests. The dimensions of the specimens are shown in Figure 2. For [0°]_20_ specimens, the width was 13 mm. Two pairs of tabs made with glass fiber-reinforced plastic were used for each specimen to reduce stress concentrations. The sample was loaded using a computer-controlled electronic universal testing machine CMT5105 of Metis (MTS) Industrial Systems (Shanghai, China) Co., Ltd., at a loading rate of 2 mm/min. For the convenience of comparison, the strength of samples with spliced structure was defined by referring to the tensile strength, the ultimate tensile load divided by the area was defined as the tensile strength.

**Figure 3:**
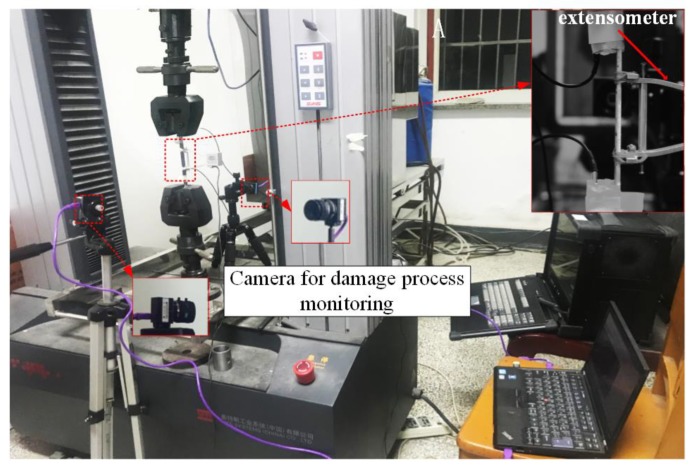
Experimental equipment setup.

During each tensile test, two cameras were used. For the convenience of analysis of the strain evolution, a macro lens was focused on one side surface that had been sprayed black and white near the splicing area. The other camera was focused on the other side surface of the specimen that had been painted white to record the damage process. The experimental device is shown in Figure 3.

## 3. Finite Element Model (FEM) Analysis/Progressive Damage Model of Composite with Ply Splices

**Figure 4:**
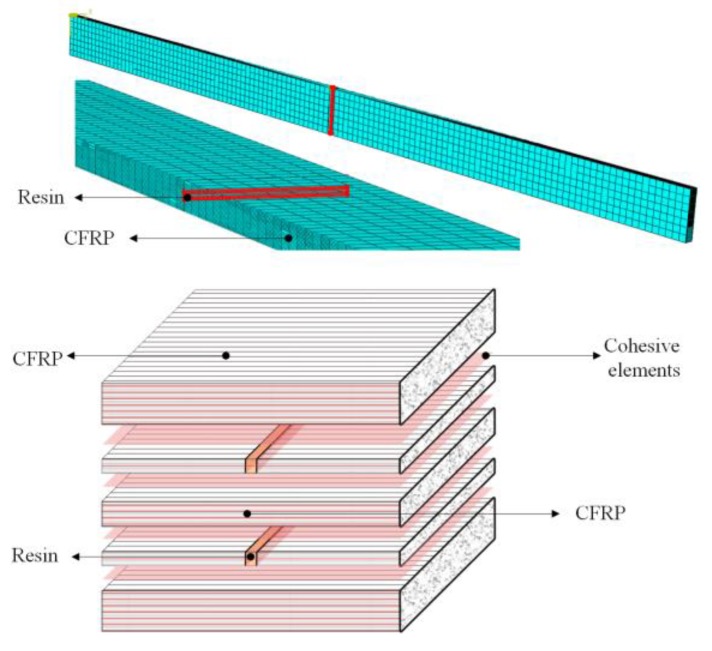
Finite element model (FEM) and splicing structure details.

A progressive damage model and cohesive zone model for these unidirectional CFRP laminates were formulated in terms of damage initiation and damage evolution using ABAQUS finite element analysis software (version 2018, Dassault SIMULIA Company, Providence, RI, USA). This research aimed to analyze the failure mechanism of samples with ply splices and an interface layer; thus, a cohesive zone model was introduced into this damage model. Each layer was simulated using a 3D deformable solid model with a C3D8R element and a thickness of 0.12 mm. The cohesive elements (COH3D8) were set to simulate the interlaminar properties between adjacent layers. The thickness of the cohesive elements was 0.001 mm. The details of finite element model and splicing structure are shown in Figure 4. The solid elements and cohesive elements connected in the form of common nodes. The cohesive elements referenced resin materials. In order to avoid interference between laminates after delamination damage, the friction contact was set on the surface of continuous fiber layer and interface layer. The material parameters are shown in Table 1. The numerical models were solved by ABAQUS Explicit, which was consistent with the experimental specimen. U_x_, U_y_ and U_z_ on the end face and U_y_ and U_z_ on the other end face were constrained to simulate the fixed-supported boundary. Meanwhile, a tensile displacement load was applied in the x direction to simulate the quasi-static tensile load.

### 3.1. Damage Initiation

Due to the prediction results usually having high accuracy, and the required parameters being easily obtained, 3D-Hashin criterion [26,27] was adopted as the failure criterion in this model. Four distinct failure modes were considered: fiber tensile failure, fiber compression failure, matrix tension failure, and matrix compression failure. The damage initiation criteria are formulated as follows.

For fiber tension failure ($\varepsilon_{11}\geq 1$):

(1) $$F_{f}^{t}={\left(\frac{\varepsilon_{11}}{X_{T}^{\varepsilon }}\right)}^{2}+{\left(\frac{\varepsilon_{12}}{S_{xy}^{\varepsilon }}\right)}^{2}+{\left(\frac{\varepsilon_{13}}{S_{xz}^{\varepsilon }}\right)}^{2}\geq 1.$$

For fiber compression failure ($\varepsilon_{11}<1$):

(2) $$F_{f}^{c}=\frac{\left|\varepsilon_{11}\right|}{X_{C}^{\varepsilon }}\geq 1.$$

For matrix tension failure ($\varepsilon_{22}+\varepsilon_{33}\geq 1$):

(3) $$F_{m}^{t}={\left(\frac{\varepsilon_{22}}{Y_{T}}\right)}^{2}+{\left(\frac{\varepsilon_{12}^{}}{S_{xy}}\right)}^{2}\geq 1.$$

For matrix compression failure ($\varepsilon_{22}+\varepsilon_{33}<1$):

(4) $$F_{m}^{c}={\left(\frac{\varepsilon_{22}}{2S_{xy}}\right)}^{2}+\left[{\left(\frac{Y_{C}}{2S_{xy}}\right)}^{2}-1\right]\frac{\varepsilon_{22}}{Y_{C}}+{\left(\frac{\varepsilon_{12}}{S_{xy}^{}}\right)}^{2}\geq 1.$$

where *F^t^_f_*, *F^c^_f_*, *F^t^_m_*, and *F^c^_m_* are failure indices corresponding to each damage mode. In Equations (1)–(4), *ε_ij_* (*i*, *j* = 1, 2, 3) is the effective stress tensor. The first subscripts, 1, 2, and 3, indicate the fiber axial direction, the in-plane transverse direction, and the out-of-plane direction, respectively. When the stress state of an element made one of the four failure indices larger than 1, the corresponding damage mode was initiated in this element and constitutive laws determined how the material entered the stage of damage evolution. *X_T_*, *X_C_*, *Y_T_*, *Y_C_*, *S_xy_*, and *S_xz_* are the axial tensile strength, axial compressive strength, transverse tensile strength, transverse compressive strength, longitudinal shear strength, and transverse shear strength of the fiber bundle, respectively.

### 3.2. Damage Evolution

An ABAQUS/VUMAT subroutine (version 2018, Dassault SIMULIA Company, Providence, RI, USA) was written to reduce the stiffness of the failure of the element. In this paper, the linear degradation model was adopted in the progressive failure model. For the damage evolution behavior, the Hillerborg [28] fracture energy theory was adopted to analyze the laminate damage process. A characteristic element length of l was introduced into the damage evolution expression, aiming to avoid the dependence of the failure behavior on the element scale and to approximate the feature length using the VUMAT embedded function charLength [28]. There were two stages: (1) the material behavior responded according to the stress–strain curve before the initiation of material damage; and (2) the material behavior responded according to the stress–displacement curve after the initiation of damage. This relationship can be described as follows:

(5) $$\varepsilon_{f,i}^{t}=\frac{2G_{i,C}^{t}}{\sigma_{t}l}$$

where *l* is the characteristic length of element, which calculates by extracting the cubic root of the volume of each element. where *G_C_* is the fracture energy required per unit area of crack growth, *σ_t_* and *ε^t^_f,i_* are the equivalent peak stress and equivalent failure strain of the tensile fracture element, respectively. *G^t^_i,C_* is the critical value of the strain energy release rate.

Here, the failure strain in the tensile mode of each element can be obtained through Equation (5), and the other failure modes were similar. For the four modes of fiber tensile failure, including fiber compression failure, matrix tension failure, and matrix compression failure, the damage state variables are defined as follows:

(6) $$d_{11}^{t}(\varepsilon_{11})=\frac{\varepsilon_{f,1}^{t}}{\varepsilon_{f,1}^{t}-\varepsilon_{0,1}^{t}}\left(1-\frac{\varepsilon_{0,1}^{t}}{\varepsilon_{11}}\right),$$

(7) $$d_{11}^{c}(\varepsilon_{11})=\frac{\varepsilon_{f,1}^{c}}{\varepsilon_{f,1}^{c}-\varepsilon_{0,1}^{c}}\left(1-\frac{\varepsilon_{0,1}^{c}}{\varepsilon_{11}}\right),$$

(8) $$d_{22}^{t}(\varepsilon_{22})=\frac{\varepsilon_{f,2}^{t}}{\varepsilon_{f,2}^{t}-\varepsilon_{0,2}^{t}}\left(1-\frac{\varepsilon_{0,2}^{t}}{\varepsilon_{22}}\right),$$

(9) $$d_{22}^{c}(\varepsilon_{22})=\frac{\varepsilon_{f,2}^{c}}{\varepsilon_{f,2}^{c}-\varepsilon_{0,2}^{c}}\left(1-\frac{\varepsilon_{0,2}^{c}}{\varepsilon_{22}}\right).$$

where the subscript 1 and 2 denote the fibre and transverse direction, respectively; *ε^t^_f,i_* and *ε^c^_f,i_* are tensile and compressive strain for damage initiation. For the tensile mode, the material fails completely in the *i* direction when *d^t^_ii_* (*ε_ii_*) = 1. For the compression mode, since the elements still have residual bearing capacity after fiber fracture, it is necessary to control the compression damage state variable between 0 and 1. In this paper, discontinuous parameter degradation reference [29]. The fiber reduction coefficient was 0.83 and the matrix reduction coefficient was 0.75.

### 3.3. Cohesive Zone Model for Interface

Interface is the bridge between the fiber bundle and the matrix, which determined how stresses were transferred. The damage status of the interface significantly influences the damage initiation and propagation of composite materials [30]. Cohesive zone modeling can be used to create a detailed simulation of the cementation, including the properties of the cementing material, and directly control the contact interface elements. The responses of cohesive elements are governed by a typical bilinear traction–separation law, and a quadratic nominal stress criterion is used to describe interfacial damage initiation. Besides, a power law criterion is adopted, which claims that failure under mixed-mode conditions is governed by a second-order power law interacting of the energies required to cause failure in the individual (normal and two shear) modes. 

**Figure 5:**
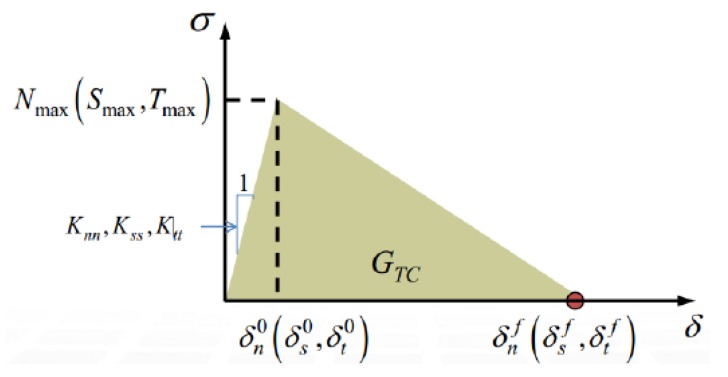
Traction–separation curve.

A typical linear traction-separation model used for fracture Modes I, II and III is shown in Figure 5. Initially, the linear elastic response is represented using the stiffness *Ki* (*i = n, s, t*). Where δ*^f^_m_* is the mixed-mode displacement at complete failure and δ^0^*_m_* is the effective displacement at damage initiation. *N_max_*, *S_max_*, and *T_max_* represent the max interface strength in normal and the two shear directions. *G_TC_* is the area enclosed by curve and coordinate axis, which is called cohesion energy. The quadratic nominal stress criterion for damage initiation and a power law criterion for failure are represented in Equations (10) and (11):

(10) $${\left\{\frac{\tau_{1}}{N}\right\}}^{2}+{\left\{\frac{\tau_{2}}{S}\right\}}^{2}+{\left\{\frac{\tau_{3}}{T}\right\}}^{2}=1,$$

(11) $${\left(\frac{G_{I}}{G_{IC}}\right)}^{\alpha }+{\left(\frac{G_{II}}{G_{IIC}}\right)}^{\alpha }+{\left(\frac{G_{III}}{G_{IIIC}}\right)}^{\alpha }=1,$$

where *τ*_1_ denotes the traction normal stress, and *τ_2_* and *τ_3_* denote shear stresses. *N*, *S*, and *T* represent the interface strength in normal and the two shear directions. Similarly, *G_I_*, *G_II_*, and *G_III_* refer to the work done by the traction and its conjugate relative displacement in the normal, first, and second shear directions, respectively. *G_IC_*, *G_IIC_*, and *G_IIIC_* are the critical fracture energies required to cause failure in each of the three directions. When Equation (11) was met, the interface element was completely destroyed, and the upper and lower interfaces were separated. Table 2 presents the interface properties, and the values of interface strengths, fracture toughness and stiffness. 

## 4. Results and Discussion

### 4.1. Failure Models

**Figure 6:**
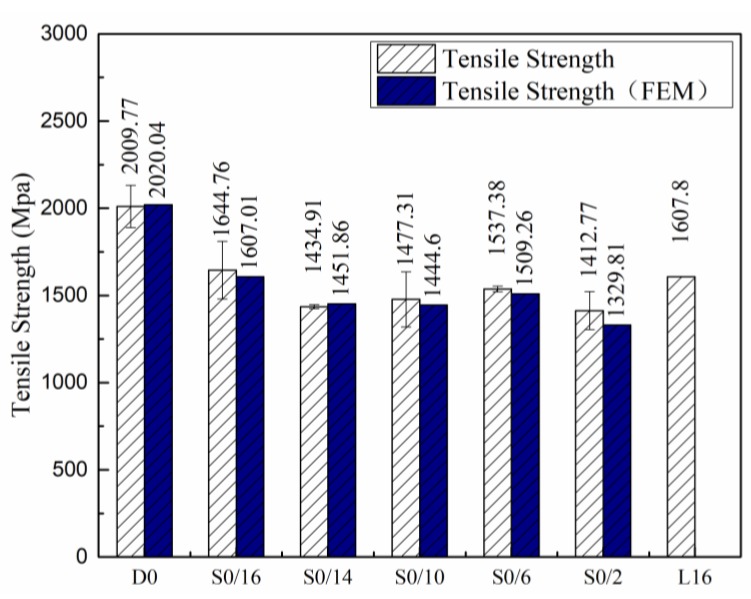
The tensile strengths of samples.

Figure 6 shows the strength of the D0, S0/16, S0/14, S0/10, S0/6, and S0/2 samples. The abscissa shows the number of splicing layers. The strength of the normal CFRP specimen (D0) was 2.0 GPa. According to our research, the tensile bearing capacity at 0° was close to the total bearing capacity of the continuous layers when the ply splice was in the same place. The strength of the sample with 20 continuous layers was converted into that of the sample with 16 continuous fiber layers, denoted as L16, that is, 2009.77 Mpa ÷ 20 × 16 = 1607.8 Mpa. The strengths of the five samples were then compared with that of L16.

Figure 6 shows the strengths of the five samples compared with that of L16. When the two ply splices were located on the surface, the tensile strength (S0/16) was the closest to L16. When the two-ply splices were inside and close to the edge, there was no significant difference in the tensile strength (S0/14 and S0/10), and their strength was slightly lower than that of L16. When the two-ply splices were evenly distributed inside the material, the tensile strength was high (S0/6). However, the tensile strength was low if the ply splices were close to each other.

**Figure 7:**
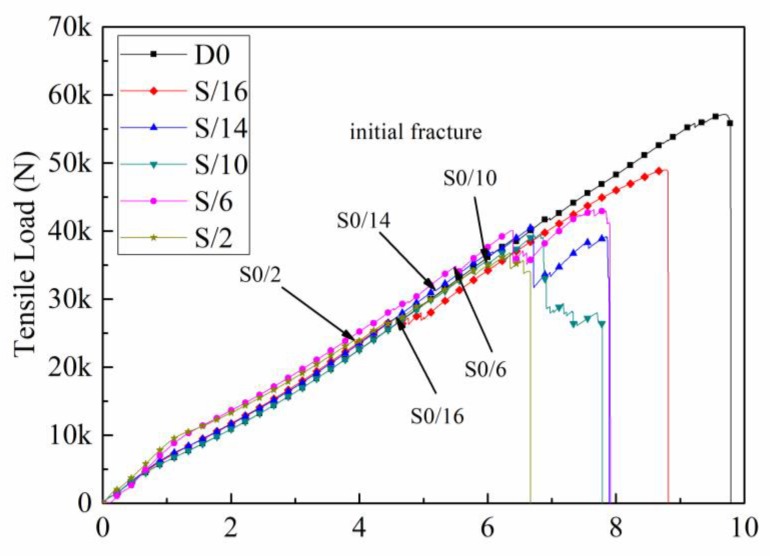
Load–displacement curves of different ply splice structures.

Figure 7 shows the load–displacement curves of the six samples in the tensile process, where the displacement represents the chuck displacement. The initial failure load of the five samples with ply splices was about 30 kN, and there was an obvious fluctuation that rose to its highest point in an approximately linear manner. As can be seen in Figure 7, the failure processes of the five structures all had three sub-processes: initial failure, crack growth, and complete failure.

**Figure 8:**
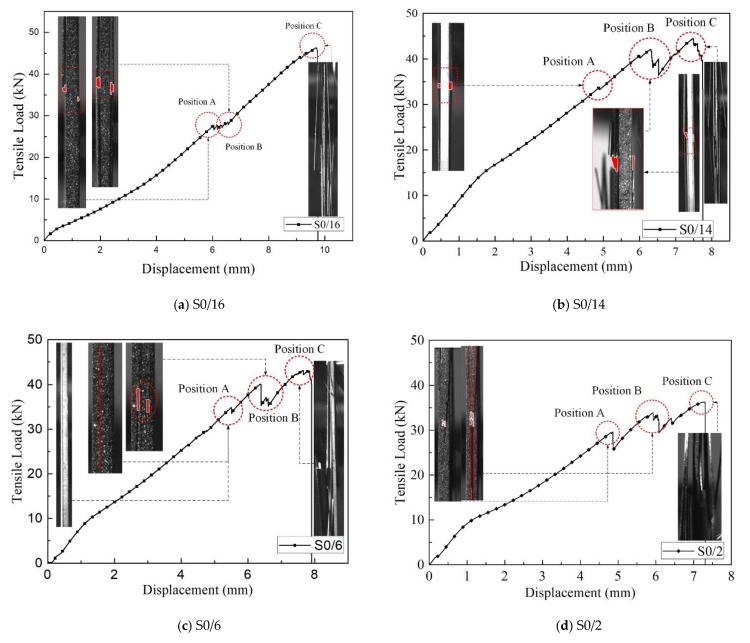
The fracture process of (**a**) S0/16; (**b**) S0/14; (**c**) S0/6; and (**d**) S0/2.

Figure 8 shows the failure process of the four samples with slightly different details. The differences were mainly caused by the different positions of the two ply splices in each sample. The failure process of S0/16, in which the splicing layers were placed on the surface, is shown in Figure 8a. When the load increased, the layers of ply splices on the surface caused delamination damage and then peeled off from the plate one by one, as shown by the several drops in the curve (Position A). After this, the curve increased linearly, and then a final break resulted when the load reached the strength of the specimen. S0/14 illustrated the case when ply splices were under the surface or near the edge, as shown in Figure 8b. The continuous fibers at the edges first broke under increasing tensile load, i.e., the first drop on the load–displacement curve (Position A). With the load increased further, four splicing layers were exposed and peeled off, similar to S0/16 at Position B. Finally, the specimen was completely destroyed when the ultimate load was reached (Position C). S0/6 shows the destruction process when two ply splices were evenly distributed in the sample (Figure 8c). As deformation increased, when the load came to Position A, intimal fracture occurred at two junction points, each leading to a through hole between the spliced two parts and delamination damage between the continuous layers and the two spliced structures. At the same time, the load decreased a little, which is shown by the first drop on the load–displacement curve. Following the initial fracture, delamination damage between the nether part of the spliced structure and the continuous layers occurred. The load similarly decreased a little, which is shown by the second drop on the curve (Position B). S0/2 typified the sample failure process when two ply splices were very close to each other as shown in Figure 8d. When the load approached Position A, the continuous fibers between the two splicing points break in advance of delamination. Following the initial fracture, delamination damage between the nether part of the spliced structure and the continuous layers occurred. The crack then expanded rapidly, and a large number of adjacent continuous fibers split (Position B) until the final break occurs (Position C). In order to make it clearer, bold red lines were used to highlight the layered cracks and holes in Figure 8. As S0/10 was in a critical condition, the experimental data were relatively discrete, and the failure mode was determined according to whether the three layers of continuous fibers outside the spliced point were damaged.

To summarize, there were two kinds of disruption modes: failure mode I was when delamination damage was followed by fiber breakage, which was similar to the failure process of samples with a single splicing point; failure mode II was defined by delamination damage after local continuous fiber failure. Comparison of the intensity results in Figure 6 shows that failure mode II had a lower tensile strength.

### 4.2. Failure Mechanism

Figure 6 compares the numerically predicted and experimentally measured effective strengths of the six kinds of samples. It was evident from Figure 6 that the FE model predicted effective strength values that were in good agreement with experimental values. Table 3 and Table 4 show the comparison between the simulated values and the experimental mean values of the initial damage load and ultimate load. It was clear from Table 3 and Table 4 that the error value was less than 10%. Considering the size effect in these experiments, the coincidence degree was high. Our results indicated that the FE model could well predict the tensile strength, the initial damage load, and the ultimate load, demonstrating the accuracy and applicability of this modeling scheme.

**Figure 9:**
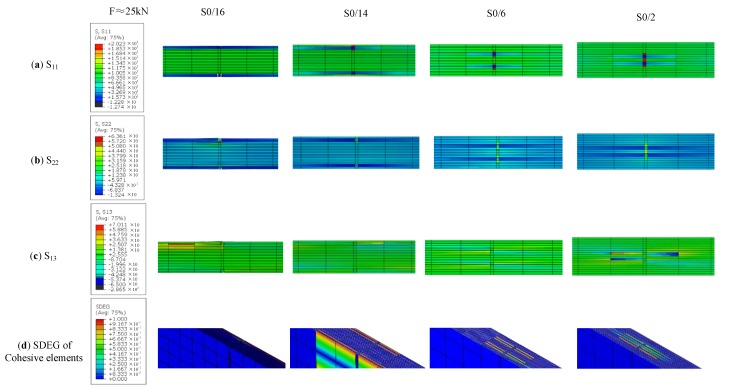
Tensile stress, transverse tensile stress, shear stress image, and damage evolution for cohesive elements of S0/16, S0/14, S0/6, and S0/2. (**a**) tensile stress S_11_; (**b**) transverse tensile stress S_22_; (**c**) shear stress S_13_; and (**d**) SDEG of cohesive elements.

**Figure 10:**
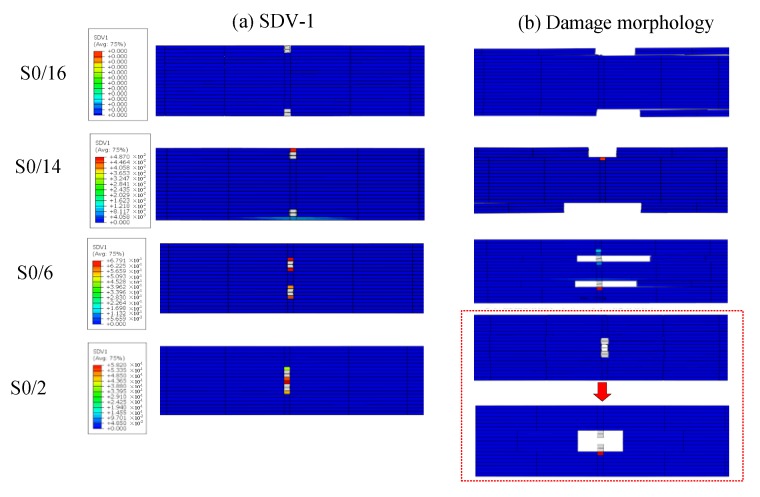
Damage evolution state of fiber elements; and the damage morphology of the ply splices in S0/16, S0/14, S0/6, and S0/2. (**a**) SDV-1; (**b**) Damage morphology.

The progressive damage process was then studied using an FEM model, as shown in Figure 4. The model was used to analyze progressive damage. In computing models, the damage strength of the laminated plates and cohesive elements were defined. Figure 9 shows the damage evolution state and stress cloud diagram of a ply splice at the moment when none of the four samples began to damage in the tensile process of S0/16, S0/14, S0/6, and S0/2 (F ≈ 25 kN). (a), (b), and (c) were, respectively, the tensile stress, the transverse tensile stress, and the shear stress nephograms of the whole structure. (d) was the stiffness degradation rate (SDEG) value distribution nephogram of cohesive elements. The damage status corresponded to the value of the particular damage variable ranging from 0 to 1, where a value of 0 indicates that damage had not occurred yet, while a value of 1 indicates that the elements were totally damaged. Figure 10 shows the damage state for fibers elements (DmgFiberT, SDV1) at the moment before the failure occurred with holes and the damage morphology after the failure of ply splices in S0/16, S0/14, S0/6, and S0/2. When the SDV1 was greater than 0, it indicated that tensile damage began to occur in the fiber direction. Table 5 lists some of the key parameter values calculated from these FEM models.

The failure mechanism of the above four samples is described below. First, we compared the stress cloud diagram values of S0/16 in Figure 9 with the strength parameters in Table 1. We saw that the value of S_22_ had reached 1.5 times the strength required by the failure, but S_11_ had not yet reached the tensile strength, which further indicated the peel failure caused by S_22_. At this moment, no fiber tension damage had occurred at these stress levels, as shown in Figure 10. Therefore, because of boundary effects, the tensile stress converted to transverse tensile stress, and the splicing layer of S0/16 peeled off mainly under the action of S_22_. For S0/14, the outermost continuous fiber of the sample first broke under the stress concentration, and then peeling off occurred with the increasing load, similar to S0/16. Therefore, the ultimate strength of S0/16 was the closest to that of L16, and the ultimate strength of S0/14 was about 87.5% of that of L16. The distribution of internal damage inside the S0/6 samples revealed that there was a little fiber tension damage occurring at these stress levels, while matrix tension damage (matrix cracking in fiber bundles) was observed in the ply splice areas. There was a certain distance between the two ply splices, so the two stress fields did not affect each other. Damage quickly extended to the cohesive elements between the continuous layers and splicing layers, indicated as two H-shaped red areas in Figure 9d. The stress distribution is shown in Figure 9c,d). From Table 5, the maximum value of S_11_ for S0/6 occurred in the continuous fiber portion near the ply splices, which was much lower than the tensile strength of the fiber direction, indicating that the continuous fiber could not be damaged. However, the maximum value of S_13_ exceeded the shear strength required for failure. This indicated that the shear component of stress in the tensile process led to the initial delamination damage of the composite, resulting in two holes that are shown in Figure 10b. In the process of interlaminar damage, it is inevitable that a few continuous fiber breaks occur near the splicing layer. Therefore, the ultimate strength of S0/6 was slightly lower than that of L16. In contrast, S0/2 was different. Under the action of tensile load, both splicing points produced stress concentrations. Due to the close distance, coupling affected the continuous fiber area in the middle of the two ply splices and facilitated stress superposition, where the SDV-1 damage value was close to 1, allowing complete destruction as shown in Figure 9b. The maximum value of S_11_ was much higher than the tensile strength required for failure. Meanwhile, S_13_ was less than the shear strength required for failure, as shown in Figure 9c,d. These results indicated that the tensile stress component of stress leads to the complete failure of the continuous fiber area in the middle of the two ply splices of S0/2, resulting in a large hole and delamination damage. The failure mechanism of S0/16 and S0/6 was consistent, and their strengths were significantly lower than that of L16.

### 4.3. Reinforcing Method for Ply Splices at Different Positions

Each layer containing a ply splice is the most common form of large-scale composite material structure or complex composite material structure. In order to avoid the impact of coupling effects, the distance of misalignment seams between the splicing positions can be widened as far as necessary if conditions are permitted. However, it is impossible to avoid the boundary effect of splicing layers near edges and the damage of independent splicing layers. These will lead to the premature destruction of a composite structure, which has a great impact on the safety and service life of a composite structure. From these aspects, the structural reinforcement of CFRP with ply splices should be carried out to provide new solutions for practical engineering problems. Therefore, when splicing is located at or near an edge, the adhesive method using composite patches at the outer surface of ply splices should be considered in order to improve local stress conditions. It is worth considering that this method is not applicable when the splicing layers are distributed independently within a sample without mutual influence or when they are close to each other. A large number of studies have shown that the interface toughness can greatly affect the stress concentration effect caused by splicing paving [31,32,33,34], which has a significant impact on mechanical properties and failure processes. Simultaneously, ply splicing can also enhance the interface performance of an adhesive layer at a splicing point and the composite patch, which also has an important influence on the reinforcing effect. Therefore, different reinforcement measures should be taken in different situations.

#### 4.3.1. For Ply Splices on or Near Edges

**Figure 11:**
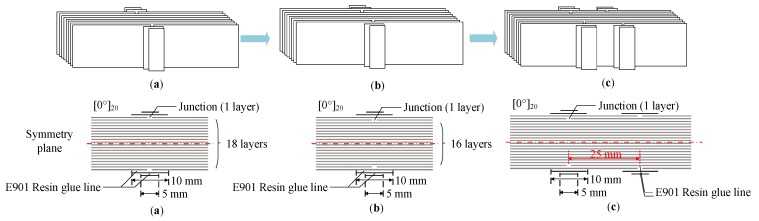
Details of double-sided reinforced samples. (**a**) S1 with patches; (**b**) S2 with patches; (**c**) S3 with patches.

The method of using reinforcement with double surface compound patches was studied by numerical simulation to determine its effect on the tensile properties of composite materials with ply splices. Three kinds of samples without reinforcement were designed, as shown in Figure 11. In the first sample, a ply splice with a layer was placed symmetrically on the surface. The second sample had a ply splice with a layer placed symmetrically on the lower layer of the surface, while in the third sample, two ply splices with a layer were placed symmetrically on the surface and the lower layer of the surface. The two splicing points were staggered, with a horizontal distance of 25 mm. The three samples were labeled as S1, S2, and S3. Two patches were attached on the outside of the ply splices, and the width was gradually reduced [35,36]. The widths were 10 mm and 5 mm. The patches and intermediate adhesive layer were made of carbon fiber and resin, similar to the above samples, whose parameters are shown in Table 1 and Table 2.

**Figure 12:**
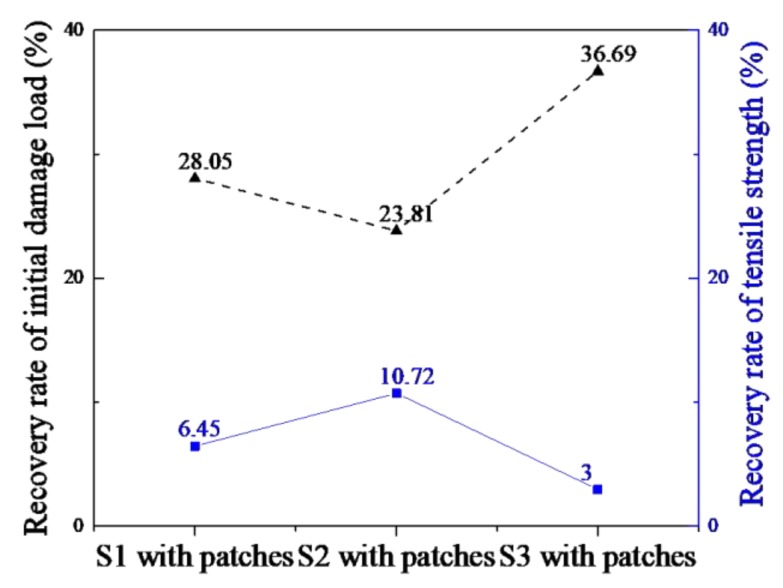
Reinforcing coefficients of tensile strength and initial damage load of three samples after double-sided reinforcement.

Table 6 presents a comparison of the initial damage load and ultimate load of the three samples calculated by numerical simulation before and after double-sided reinforcement. Figure 12 shows the reinforcing coefficients of tensile strength and initial damage load of three samples after double-sided reinforcement. The reinforcing coefficient can be calculated according to the following Equations:

(12) $$\eta_{f}=\frac{F_{D}^{r}-F_{D}}{F_{D}}\times 100\%$$

(13) $$\eta_{\sigma }=\frac{\sigma_{t}^{r}-\sigma_{t}^{}}{\sigma_{t}^{}}\times 100\%,$$

where *η_σ_* and *η_f_* are the reinforcing coefficient of strength and initial damage load respectively; *F_D_^r^* is the initial damage load of the sample with double-sided patches, N; *F_D_* is the initial damage load of samples without reinforcement, N; *σ_r_^t^* is the tensile strength of the sample with double-sided patches, MPa; and *σ_t_* is the tensile strength of samples without reinforcement, MPa.

The numerical simulation results of S1 and S2 without reinforcement were consistent with the experimental results of S0/16 and S0/14. S1 was a first interlaminar shear stratification mode, and S2 was first destruction and then stratification mode. As can be seen from Table 6 and Figure 12, when a patch was added on both sides, the failure mode changed from Mode I and Mode II to Mode III. This indicated that double-sided patch reinforcement could effectively prevent the early fracture of continuous fibers on the surface and delay surface peeling. This led to the initial damage load of the ply splices of the three samples being greatly improved up to 37% and the ultimate load being increased to a small degree. Thus, the tensile properties of composites with ply splices can be effectively improved using patches. Our results show that the repair effect of patching on different splicing layers was different.

**Figure 13:**
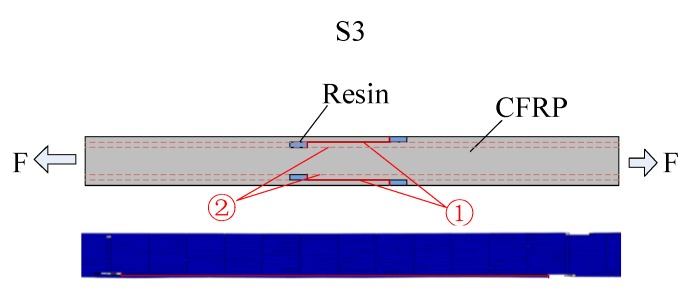
Failure mechanism for S3.

The failure process of S3, which was a sample without reinforcement, is shown in Figure 13. Due to the coupling effect after misalignment of the seams of the two splicing structures, stress concentration occurred at both points. At this time, the stress strength was relatively low, so it did not lead to external fiber pre-failure. This also explained why the initial damage load of the ply splice with misalignment seams was higher than that of S0/16. In the process of tension, with increasing load, the splicing layer on the surface was first destroyed due to the low interface strength of the resin and fiber adhesive layer. Simultaneously, stress was redistributed. In Position ① of a large shear stress and an occurrence of layer, as the stress increased, shear failure occurred in Position ②, and z-shaped cracks appeared, as shown in Figure 13.

When double-sided patches were added to the samples, under the action of the tensile stress in the length direction, the stiffness at the repair position of the patch increased, which was useful for delaying damage. This also had a good impact on S3, leading to delay in the initial damage load because there were two patches. With continuous increase in the load, micro-bending deformation between the patches and parent plate changes the tensile stress from the length direction (S_11_) to the thickness direction (S_22_) and plays a reinforcing role. Due to the low strength of composite laminates in the direction of thickness and the high strength of the matrix, the tensile stress in the direction of thickness finally leads to surface peeling failure of the parent plates. However, S3 had peeling stress in two positions. Under the coupling effect, when one patch was peeled off, the other patch follows, and then the fibers with ply splices on the surface quickly undergo delamination damage and then are completely destroyed, resulting in a relatively low overall repair effect on the tensile strength.

#### 4.3.2. For Independent or Closely Bundled Ply Splices Inside a Composite

**Figure 14:**
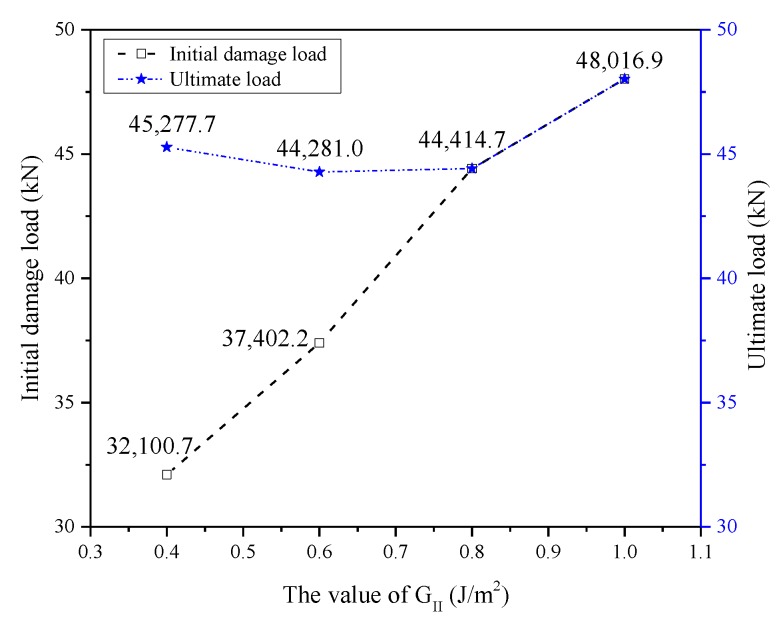
Initial damage load and ultimate load for different values of *G_II_*.

Fracture toughness is one of the important factors affecting interfacial toughness. Under the action of tensile load, a fiber breaks gradually with matrix cracking or interfacial debonding (interfacial debonding was considered a type II crack along the fiber/matrix interface). Therefore, the fracture energy *G_II_* has a great influence on the tensile properties of composite materials containing splicing layers. As shown in Figure 14, when the interface strength was consistent, the value of *G_II_* increased gradually to change the bond degree of the interface, which was 0.4 J/m^2^, 0.6 J/m^2^, 0.8 J/m^2^, and 1.0 J/m^2^. According to the analysis in Figure 14, when the *G_II_* value increased to a certain extent, the initial damage was delayed, and finally, the failure mode changed from delamination damage and then destruction to simultaneous destruction of the matrix and fiber, which was the same as the failure mode after double-sided patch reinforcement. The stiffness of an interface decreased with increasing toughness, leading to a gentler stress concentration at the interface and more energy consumed during delamination damage. Thus, interface stratification appeared later in the damage process. When the interfacial toughness was weak, the damage of composites was basically axial interfacial shear debonding caused by shear stress at the interface between fiber and matrix. On the contrary, when the interfacial toughness was strong, the damage at the splice extended to the matrix, and the direction was perpendicular to the axial direction, causing both interfacial delamination damage and matrix fracture, which was perpendicular to the fiber axial matrix fracture. In summary, an increase in the interfacial toughness can effectively improve the tensile strength of composites with ply splicing, especially with a gradual increase in the initial damage load.

## 5. Conclusions

The tensile properties of unidirectional carbon fiber-reinforced composites with ply splices were tested and analyzed using a three-dimensional FE model with continuum damage and cohesive zone models. The proposed model was in good agreement with experimentally determined load and strength values. Based on the above results, the following conclusions can be drawn.

1. Based on the proposed failure mechanism of unidirectional CFRP splicing with two laminates in different locations, we found that when ply splices were located on the surface, a sample underwent peel failure under the stress of S_22_; when ply splices were close to the surface, the continuous fibers on the outside of a sample broke first and then peel failure occurred under the stress of S_22_; when ply splices were independent of each other, the delamination damage of the two splicing points occurred under the shear stress S_13_ before the local fiber fracture; and when ply splices were close to each other, leading to early fracture of the continuous fibers between them under the tensile stress S_11_, delamination damage occurred. In general, the tensile strengths of samples with continuous fibers that break first were generally lower. When the fibers did not break first, the tensile strengths of samples were equivalent to that of continuous fibers.
2. Some reinforcing methods were proposed. When the ply splices located or near the edge were reinforced by double-sided patches, the initial damage load increased by about 40%, and the tensile strength increased by about 10%. When the ply splices that were independent of each other were reinforced by increasing the interfacial toughness of the adhesive layer, the initial damage load increased by about 50%, and the tensile strength increased by about 5%. The tensile strength was enhanced by the two methods, especially the initial damage load was greatly increased.

Based on the above conclusions, some suggestions could be made for material design when ply splice structures are involved: high fracture toughness resin can relieve the stress concentration caused by the ply splice; the ply splicing positions should keep as far away with each other as possible to avoid coupling effects; when the ply splices are designed on and near the surface, surface enhancements using additional patches on the ply splicing position will be helpful to increase the strength. 

## Figures and Tables

**Table 1:** Material properties and strength parameters of T300/ E901 composites.

Material	Mechanical Parameters	Value
E901 epoxy resin	E/Gpa	3.78
	v	0.35
Unidirectional CFRP	E_1_/Gpa	127.34
	E_2_/Gpa	7.78
	E_3_/Gpa	7.78
	ν_12_	0.27
	ν_13_	0.27
	ν_23_	0.42
	G_12_/Gpa	5.00
	G_13_/Gpa	5.00
	G_23_/Gpa	3.08
	X_t_/MPa	2114
	X_c_/MPa	704
	Y_t_/MPa	80
	Y_c_/MPa	68
	S_12_ = S_13_/MPa	80
	S_23_/MPa	55

Note: E is Young’s modulus; ν is Poisson’s ratio; G is the shear modulus; X is the longitudinal strength; Y is the lateral strength; Subscripts 1, 2, and 3 represent directions 1, 2, and 3, respectively. Subscript t represents tension; Subscript c represents compression.

**Table 2:** The values of interface strengths, fracture toughness and stiffness.

*N*_max_ (MPa)	*S*_max_ = *T*_max_ (MPa)	*G_IC_* (mJ/mm^2^)	*G_IIC_* = *G_IIIC_* (mJ/mm^2^)	*K_nn_*/10^−6^	*K_ss_* = *K*_tt_/10^−6^
54.6	60	0.2	0.4	3.78	1.4

**Table 3:** FEM values of initial damage load compared with the experimental mean.

Initial Damage Load	Experimental/N	FEM/N	Error
S0/16	27,601.81	27,770.6	0.61%
S0/14	31,164.32	27,503.2	8.82%
S0/10	33,608.3	34,451	2.51%
S0/6	29,872.34	32,100.7	7.13%
S0/2	26,748.06	23,006.5	7.04%

**Table 4:** FEM values of ultimate load compared with the experimental mean.

Ultimate Load	Experimental/N	FEM/N	Error
D0	58,636.92	60,601.3	3.35%
S0/16	46,972.66	48,210.2	2.63%
S0/14	40,834.36	43,555.8	6.66%
S0/10	40,997.75	43,338	5.71%
S0/6	43,900.88	45,277.7	3.14%
S0/2	39,087.81	39,894.3	2.06%

**Table 5:** Maximum values in FEM models.

Specimen	Load/kN	CFRP			Cohesive Element *	
		S_11_/MPa	S_22_/MPa	S_13_/MPa	S_23_/MPa	S_33_/MPa
S0/16	27.7	2025	116.2	107.2	32.78	36.62
S0/14	27.5	1896	27.6	53.97	33.85	8.715
S0/6	32.1	1973	27.54	52.69	28.24	7.88
S0/2	23.0	1936	22.6	51.48	27.7	5.91

* Cohesive elements in between splicing layers and continuous layers.

**Table 6:** FEM values of initial damage load and ultimate load for samples before and after double-sided repair.

Sample	Initial Damage Load/N	Ultimate Load/N	Failure Mode
S1	40,341.1	49690.8	I
S1 with patches	51,657.8	52896	III
S2	43,966.4	49162	II
S2 with patches	54,434.4	54434.4	III
S3	36,410.1	48341.4	I
S3 with patches	49,769.1	49769.1	III

Notes: Failure mode I is delamination damage and then fiber breakage; Failure mode II is first fiber breakage and then delamination damage. Failure mode III is delamination damage and fiber breakage occurring simultaneously, resulting in overall destruction.
